# Psychometric properties of the CDC Symptom Inventory for assessment of Chronic Fatigue Syndrome

**DOI:** 10.1186/1478-7954-3-8

**Published:** 2005-07-22

**Authors:** Dieter Wagner, Rosane Nisenbaum, Christine Heim, James F Jones, Elizabeth R Unger, William C Reeves

**Affiliations:** 1 Division of Viral and Rickettsial Diseases, National Center for Infectious Diseases, Centers for Disease Control and Prevention, 1600 Clifton Rd, Mail Stop A15, Atlanta, GA, USA; 2 St Michaels Hospital, Inner City Health Research Unit, Toronto, Canada; 3 Department of Psychiatry and Behavioral Sciences, Emory University School of Medicine, Atlanta, GA, USA

## Abstract

**Objectives:**

Validated or standardized self-report questionnaires used in research studies and clinical evaluation of chronic fatigue syndrome (CFS) generally focus on the assessment of fatigue. There are relatively few published questionnaires that evaluate case defining and other accompanying symptoms in CFS. This paper introduces the self-report CDC CFS Symptom Inventory and analyzes its psychometric properties.

**Methods:**

One hundred sixty-four subjects (with CFS, other fatiguing illnesses and non fatigued controls) identified from the general population of Wichita, Kansas were enrolled. Evaluation included a physical examination, a standardized psychiatric interview, three previously validated self-report questionnaires measuring fatigue and illness impact (Medical Outcomes Survey Short-Form-36 [MOS SF-36], Multidimensional Fatigue Inventory [MFI], Chalder Fatigue Scale), and the CDC CFS Symptom Inventory. Based on theoretical assumptions and statistical analyses, we developed several different Symptom Inventory scores and evaluated them on their ability to differentiate between participants with CFS and non-fatigued controls.

**Results:**

The Symptom Inventory had good internal consistency and excellent convergent validity. A Total score (all symptoms), Case Definition score (CFS case defining symptoms) and Short Form score (6 symptoms with minimal correlation) differentiated CFS cases from controls. Furthermore, both the Case Definition and Short Form scores distinguished people with CFS from fatigued subjects who did not meet criteria for CFS.

**Conclusion:**

The Symptom Inventory appears to be a reliable and valid instrument to assess symptoms that accompany CFS. It is a positive addition to existing instruments measuring fatigue because it allows other dimensions of the illness to be assessed. Further research is needed to confirm and replicate the current findings in a normative population.

## 1. Background

Chronic fatigue syndrome (CFS) is an incapacitating illness defined by disabling chronic fatigue and characteristic accompanying symptoms [1]. CFS has no confirmatory physical signs or characteristic laboratory abnormalities and its etiology and pathophysiology remain unknown [2]. Many of the problems searching for markers of CFS reflect ambiguities of definition. Recently, an *International CFS Study Group *identified areas of vagueness in the case definition and proposed revisions in its utilization [3]. The *Group *recommended that validated standardized instruments be used to evaluate the functional impairment, fatigue, and accompanying symptoms associated with CFS. They recommended that future research utilize the Medical Outcomes Survey Short-Form-36 (SF-36) [4] to assess functional impairment and that the Checklist Individual Strength (CIS) [5] or Multidimensional Fatigue Inventory (MFI) [6] be used to measure general dimensions of fatigue. The *Group *was unaware of standardized and validated instruments that assessed the CFS symptom complex and suggested that investigators consider using a symptom checklist developed by the US Centers for Disease Control and Prevention (CDC) for use in population-based surveys. The CDC Symptom Inventory assesses the full range of CFS associated symptoms, has been used in several population-based studies (i.e., comparative data are available), and is publicly available. However, the Symptom Inventory has not been formally validated. The objective of the present study was to validate the Symptom Inventory as a questionnaire for evaluation of CFS associated symptoms.

## Methods

### Study design/subjects

This study adhered to human experimentation guidelines of the U.S. Department of Health and Human Services and the Helsinki Declaration. The CDC Human Subjects committee approved study protocols. All participants were volunteers who gave informed consent.

This study enrolled subjects who had previously participated in the 1997 through 2000 Wichita CFS Surveillance Study [7,8]. In brief, the *Surveillance Study *used a random-digit-dialing telephone survey to screen 56,146 adult residents (18 to 69 years of age) of Wichita, Kansas. A surveillance cohort of 3,528 adults who reported fatigue of at least 1-month duration and 3,634 non-fatigued persons completed a detailed telephone interview and eligible subjects were clinically evaluated to assess CFS. A subset of the cohort was followed at 12-, 24-and 36-months with a telephone interview and clinical evaluation. Fatigued participants in the present study were a subset of the 659 fatigued adults identified during surveillance who were classified as CFS by 1994 research case definition criteria [1] or unexplained chronic fatigue not meeting criteria for CFS (ISF). Non-fatigued controls were randomly selected from the cohort who participated in all telephone interviews at baseline, 12-, 24-, and 36-month follow-up periods, who had never reported fatigue of at least 1-month duration, and who had never been identified with medical or psychiatric conditions exclusionary for CFS.

### Clinical Measurements

People who agreed to participate were admitted to a Wichita hospital research unit for 2-days. Upon arrival, they provided a standardized past medical history, a review of current medications; they then underwent a brief standardized physical examination and standardized psychiatric evaluation of Axis I disorders (DIS) [9] that exclude classification of CFS (melancholic major depression, bipolar disorder, psychosis, substance abuse, eating disorders) [1,3]. We also collected blood and urine for routine analysis, including: a complete blood count with differential, C-reactive protein, alanine aminotransferase, albumin, alkaline phosphatase, aspartate aminotransferase, total bilirubin, calcium, carbon dioxide, chloride, creatinine, glucose, potassium, total protein, sodium, urea nitrogen BUN, pregnancy test, TSH, free T-4, and urinalysis. In addition they completed the CDC Symptom Inventory and 3 well standardized and validated self-administered questionnaires (described below). Subjects were classified based on results of laboratory, physical and psychologic examination, fatigue and symptoms at the time of the study as: CFS (currently meeting the 1994 CFS research case definition) [1]; ISF (currently unexplained fatigue but not meeting CFS criteria); remission (prior CFS or ISF but currently not fatigued); never fatigued (non-fatigued controls); or, excluded (missing data, laboratory or psychological abnormalities).

The Medical Outcomes Survey Short-Form (SF-36) [4] (QualityMetric Incorporated, Lincoln, Rhode Island) assesses function and well-being in 8 areas: 1) limitations in physical activities because of health problems; 2) limitations in social activities because of physical or emotional problems; 3) limitations in usual role activities because of physical health problems; 4) bodily pain; 5) general mental health; 6) limitations in usual role activities because of emotional problems; 7) vitality (energy and fatigue); and 8) general health perceptions. Scores in each area reflect ability to function (higher values being better).

The Multidimensional Fatigue Inventory (MFI) [6] is a 20-item self-report instrument that measures 5 dimensions of fatigue; General Fatigue, Physical Fatigue, Mental Fatigue, Reduced Motivation and Reduced Activity. The score in each dimension reflects severity of fatigue (higher values being worse). The MFI has been primarily used to measure fatigue in cancer patients [10].

The Chalder Fatigue Scale (Chalder) [11] includes 10 questions measuring physical and mental fatigue. We added 2 questions regarding muscle pain: "Do your muscles hurt at rest?" and "Do your muscles hurt after exercise?".

### CDC CFS Symptom Inventory

The Symptom Inventory collects information about the presence, frequency, and intensity of 19 fatigue and illness-related symptoms during the month preceding the interview; these include all 8 CFS-defining symptoms (post-exertional fatigue, unrefreshing sleep, problems remembering or concentrating, muscle aches and pains, joint pain, sore throat, tender lymph nodes and swollen glands, and headaches). It also catalogues diarrhea, fever, chills, sleeping problems, nausea, stomach or abdominal pain, sinus or nasal problems, shortness of breath, sensitivity to light, and depression. Perceived frequency of each symptom was rated on a four-point scale (1 = a little of the time, 2 = some of the time, 3 = most of time, 4 = all of the time), and severity or intensity of symptoms was measured on a three-point scale (1 = mild, 2 = moderate, 3 = severe).

### Symptom Inventory Scoring

To summarize the degree of distress associated with each symptom, individual symptom scores were calculated by multiplying the frequency score by the intensity score. We transformed the intensity scores into equidistant scores before multiplication (i.e., 0 = symptom not reported 1 = mild, 2.5 = moderate, 4 = severe) resulting in range 0–16 for each symptom. We calculated a Total score for each person by summing the 19 individual symptom scores (possible range from 0 to 304). We also defined a Case Definition score as the sum of the 8 individual CFS case-definition symptom scores and an Other Symptoms score by considering only the 11 non-CFS symptoms.

### Short Form of the CDC Symptom Inventory

We also explored the possibility of deriving a shorter version of the Symptom Inventory that would be a reliable and economic screening instrument. We created a Short Form of the Symptom Inventory by consecutively eliminating those symptoms whose scores had a corrected item-total score correlation < 0.60. The Short Form retained 6 symptoms: unusual fatigue after exertion, unrefreshing sleep, muscle aches, sleeping problems, problems with memory, and problems with concentration.

### Statistical Analyses

To evaluate the internal consistency of the MFI and the Symptom inventory, we performed reliability analyses based on the model of averaging the inter-item correlation. Pearson's correlation coefficients between the CDC Symptom Inventory, MFI, Chalder Fatigue Scale and SF-36 were determined to evaluate convergent validity. We assessed construct validity by using one-way analyses of variance and Bonferroni post-hoc group comparisons to compare the CDC Symptom Inventory scores across the fatigue groups. We compared the Short and Total Forms with respect to psychometric properties (i.e., internal consistency and validity). The practicability of both scores was compared with three more intuitive scores derived from the Symptom Inventory; sum of all 19 individual frequency scores (Frequency Score), sum of all 19 individual intensity scores (Intensity Score), and the number of reported symptoms.

## Results

Two hundred twenty-seven people participated in the 2-day clinical evaluation and participation rates (64 to 78%) were similar among the categories (p = .26). Five of the 227 were excluded because of incomplete psychiatric interviews, 29 because of exclusionary medical conditions, 3 because of psychiatric conditions, and 26 because of current major depression disorder with melancholic features, resulting in 164 subjects for analysis. Twenty-four subjects with prior fatigue (7 CFS and 17 ISF) were classified as in remission. There were no differences in age, body mass index, or sex between the classification groups (Table 1).

**Table 1:** Characteristics by subject classification (N = 164). BMI is body mass index, CFS includes subjects with chronic fatigue syndrome, ISF includes those with unexplained chronic fatigue not meeting criteria for CFS, and NF are never fatigued controls

	**N**	**Women (%)**	**Age (Mean ± SD)**	**BMI (Mean ± SD)**
**Classification**				
CFS	52	44 (84.6%)	49.9 ± 7.9	28.4 ± 5.1
ISF	40	29 (72.5%)	49.7 ± 9.3	28.7 ± 4.7
Remission	24	17 (70.8%)	51.2 ± 9.1	28.8 ± 5.1
Never Fatigued	48	41 (85.4%)	50.3 ± 8.5	28.9 ± 5.1

### Reliability analyses

Reliability analyses revealed good internal consistency for the reduced motivation subscale of the MFI and excellent internal consistency for the other four subscales. Cronbach's alpha coefficients were 0.89 for general fatigue, 0.82 for physical fatigue, 0.90 for reduced activity, 0.77 for reduced motivation, and 0.92 for mental fatigue. These findings are similar to those of Smets and colleagues [6].

The Symptom Inventory Total score also reflected excellent internal consistency, with a Cronbach's alpha coefficient of 0.88: Cronbach's alpha was 0.87 for the Symptom Inventory Short-Form. Table 2 shows the corrected item-total correlations (product terms) of all symptoms for the Total score and Short Form. In addition, reliability analyses revealed a Cronbach's alpha of 0.82 for the Case Definition score and 0.74 for the Other Symptoms score. Table 3 shows the descriptive data for the Total, Case Definition, Short Form, and the Other Symptoms scores.

**Table 2:** Corrected item to total correlations for the Symptom Inventory Total Score and the Symptom Inventory Short-Form Score

**Symptom**	**Corrected item to total correlations**	
	
	**Total score**	**Short-form score**
Sore throat	.43	
Tender nodes	.48	
Diarrhea	.37	
Unusual fatigue after exertion	.69	.64
Muscle aches	.70	.64
Joint pain	.54	
Feverishness	.28	
Chills	.52	
Unrefreshing sleep	.77	.79
Sleeping problems	.65	.70
Headaches	.43	
Memory problems	.62	.66
Concentration	.59	.67
Nausea	.40	
Stomach pain	.32	
Sinus problems	.51	
Shortness of breath	.41	
Sensitivity to light	.41	
Depression	.52	

**Table 3:** Descriptive data of the CDC Symptom Inventory Scores

**CDC Symptom Inventory Scores**	**Mean**	**SD**	**Min**	**Max**
Total	36.22	33.87	0	153.50
Short-form	19.51	20.01	0	96
CDC Case definition	21.21	21.54	0	102
Other symptoms	15.02	14.11	0	62

### Validity

#### Convergent validity of Symptom Inventory

The Total, Case Definition and Short Form scores all had good convergent validity as determined by correlations with the MFI, Chalder Fatigue Scale, and SF-36 subscales (Table 4). As expected, high scores on the Symptom Inventory correlated with high fatigue scores and low levels of function. In other words, subjects who scored high on questionnaires assessing fatigue and low on those assessing functioning and well being, generally had high Symptom Inventory scores.

**Table 4:** Pearson's correlation matrix of CDC Symptom Inventory scores and MFI, Chalder Fatigue Scale, and SF-36 subscales (N = 164)

	**Total score**		**Short-form**		**Case definition score**	
**Questionnaires**	**r**	**P**	**r**	**P**	**r**	**P**
**MFI**						
General fatigue	.64	< .001	.67	< .001	.63	< .001
Physical fatigue	.60	< .001	.62	< .001	.62	< .001
Reduced activity	.60	< .001	.62	< .001	.57	< .001
Reduced motivation	.53	< .001	.53	< .001	.50	< .001
Mental fatigue	.54	< .001	.56	< .001	.54	< .001
**Chalder Fatigue Scale**	.74	< .001	.76	< .001	.75	< .001
**SF-36**						
Physical functioning	-.58	< .001	-.56	< .001	-.60	< .001
Role-physical	-.64	< .001	-.56	< .001	-.62	< .001
Bodily pain	-.67	< .001	-.56	< .001	-.68	< .001
General health	-.59	< .001	-.59	< .001	-.60	< .001
Vitality	-.68	< .001	-.69	< .001	-.67	< .001
Social functioning	-.66	< .001	-.62	< .001	-.63	< .001
Role-emotional	-.39	< .001	-.40	< .001	-.37	< .001
Mental health	-.46	< .001	-.48	< .001	-. 41	< .001
**CDC Symptom Inventory Scores**						
Total			.94	< .001	.97	< .001

Short-form	.94	< .001			.95	< .001

#### Construct validity

**Figure 1:**
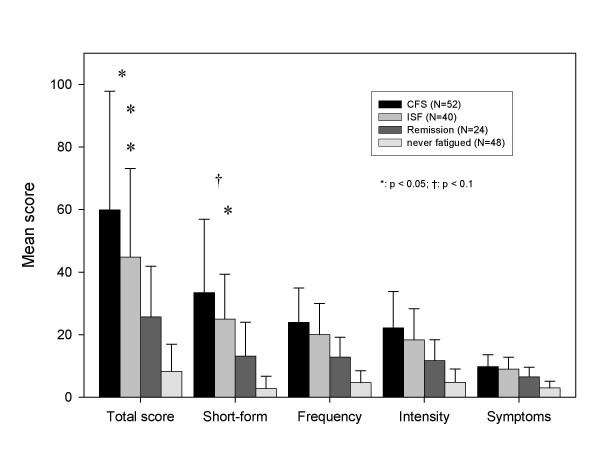
**Mean scores for Symptom Inventory by subject classification. **All post-hoc comparisons between never fatigued and CFS or ISF were significant (at least: p < 0.05). All post-hoc comparisons between Remission and CFS or ISF were significant (at least: p < 0.05).

**Figure 2:**
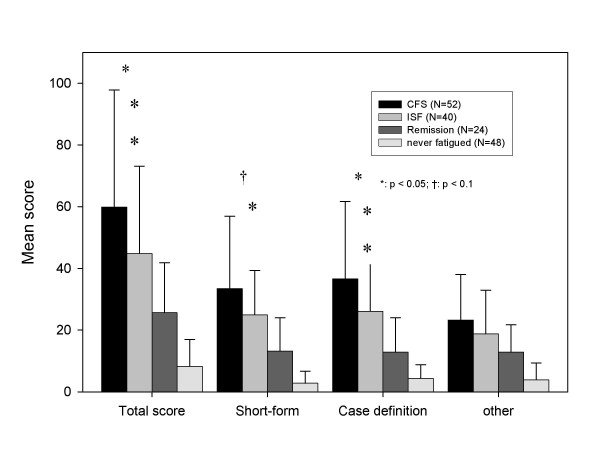
**Mean differences for the different Symptom Inventory Product Term Scores between subjects whose overall fatiguing illness history status was classified as classified as CFS, ISF, Remission and never fatigued controls. **All post-hoc comparisons between never fatigued and CFS or ISF were significant (at least: p < 0.05). All post-hoc comparisons between Remission and CFS or ISF were significant (at least: p < 0.05 – not valid for other symptoms: Ever ISF vs. Remission).

The extent to which Symptom Inventory scores discriminate between subgroups classified as to fatigue status (e.g., CFS versus not fatigued or CFS versus ISF, CFS versus remission) is one measure of the Inventory's practicability for assessing fatiguing illnesses. All Bonferroni post-hoc comparisons between never fatigued controls and those classified as CFS or ISF showed significant mean differences related to Symptom Inventory scores (Figures 1 and 2). Also, those classified as in remission had significantly lower symptom impact than those with CFS or ISF. The Total score and the Case Definition score distinguished between subjects classified CFS or ISF (Bonferroni post-hoc test; p < .05), while the Symptom Inventory Short Form revealed only a trend towards higher symptom impact for the CFS. Subjects classified as CFS and ISF were similar with respect to the Frequency score, the Intensity score, and number of symptoms.

## Discussion

This study showed that the CDC CFS Symptom Inventory is a reliable and valid instrument for assessing symptoms associated with CFS. Many studies conducted in tertiary care settings have used ad hoc (non-validated) questionnaires to assess the frequency or intensity of CFS-defining symptoms. In addition to lack of validation, one unanswered question from such studies concerns what is worse – a severe symptom that occurs sporadically or a clinically minimal symptom occurring every day. The Symptom Inventory obviates this problem because it is scored as a product-term of intensity and frequency and better represents the variance of each symptom.

Both the Total and the Case Definition scores effectively assessed initial study classification as CFS, ISF, and never fatigued. They also showed excellent psychometric properties, including good internal consistency and validity. One might question the possibility of circular logic – defining an illness by its symptoms then assessing psychometric properties of a scale that measures the same symptoms. Unfortunately, as yet, CFS has no confirmatory physical signs or characteristic laboratory abnormalities [2]; so, in lieu of a 'gold standard', it is defined by disabling chronic fatigue and characteristic accompanying symptoms [1,3]. For initial classification in this study, we defined CFS by literally applying criteria of the 1994 case definition [1]. We classified subjects as CFS who stated that they had been fatigued for at least 6-months; that the fatigue severely affected their occupational, educational, social, or recreational activities; who endorsed the presence of at least 4 CFS defining symptoms; and, who had no exclusionary medical or psychiatric conditions. Subjects who had medically/psychiatrically unexplained chronic fatigue not fulfilling all these criteria were considered ISF. This operational classification notwithstanding, CFS represents a multi-faceted illness. Fatigue is a complex construct and we employed 2 standardized and validated instruments (the MFI and Chalder Fatigue Scale) to evaluate its various dimensions (e.g., physical fatigue, mental fatigue). Similarly, the impairment associated with CFS is not unidimensional; so we utilized the SF-36 to quantify the 8 major dimensions of function and wellbeing. Finally, CFS includes a characteristic symptom complex and we used the Symptom Inventory to evaluate the intensity and frequency of accompanying symptoms. The Total, Case Definition, and Short Form scores all had good convergent validity as determined by correlations with the 3 multidimensional measures of fatigue and impairment. The Symptom Inventory scores also discriminated between subgroups originally classified as CFS, ISF or not fatigued and this is a measure of the instrument's practicability for assessing fatiguing illness.

As noted above, there is no objective test to unequivocally diagnose CFS so it is premature to evaluate sensitivity or specificity of the various Symptom Inventory scores. Rather, our objective was to evaluate the Inventory's psychometric properties as baseline for its use in future studies. Studies of the clinical characteristics of CFS and other unexplained fatiguing illnesses should utilize the Symptom Inventory in conjunction with other instruments that assess different dimensions and consequences of fatigue (e.g., the MFI, SF-36) [3,10]. Beside the Symptom Inventory Total and the Case Definition scores, the Symptom Inventory Short Form score, consisting of only 6 symptoms (unusual fatigue after exertion, unrefreshing sleep, muscle aches, sleeping problems, memory and concentration problems), appears to be an economic and precise screening measurement for the current status of fatiguing illnesses.

The Symptom Inventory includes 10 symptoms that are not used to define CFS. Although not considered in the case definition, these symptoms are commonly reported by chronically ill people and have proven useful for stratification during analysis of descriptive and case control studies. In addition, the *International CFS Study Group *recommended additional research to further develop the CFS case definition and such research must assess a comprehensive range of symptoms. Finally, analytic studies of CFS must consider somatization disorders (both as confounders and comorbid conditions); the full range of symptoms in the Inventory is needed to categorize such disorders.

At least one important limitation must be considered. Although study subjects were recruited from the community and do not reflect the strong biases inherent of clinic patient populations, they did not represent the general population; rather they comprised a sample of people with and without unexplained fatiguing illnesses. Thus, the excellent psychometric properties of the Symptom Inventory cannot be generalized to the general population. To further validate and evaluate the Symptom Inventory, additional testing in a larger population-based sample not stratified by fatigue is required. There is also a need to determine the test-retest-reliability and stability of the Symptom Inventory. The present study provides preliminary results to encourage researchers to administer the Symptom Inventory along with other standardized questionnaires measuring fatigue and functional impairment in studies of CFS and other fatiguing illnesses.

## Competing interests

The author(s) declare that they have no competing interests.

## Authors' contributions

DW conceived of the scale to summarize symptom impact, had primary responsibility for statistical analysis and wrote the manuscript; RN collaborated in study design, in data analysis and writing the manuscript; CH was instrumental in the conception and design of the study, participated in fieldwork, collaborated in analysis and interpretation of the data, and writing the manuscript; JFJ collaborated in the clinical study and in preparation of the manuscript; ERU was instrumental in the conception and design of the study, collaborated in interpretation of the data, and writing the manuscript; WCR conceived of the study, served as principal investigator throughout its execution and collaborated in writing the manuscript.
